# Etracker: A Mobile Gaze-Tracking System with Near-Eye Display Based on a Combined Gaze-Tracking Algorithm

**DOI:** 10.3390/s18051626

**Published:** 2018-05-19

**Authors:** Bin Li, Hong Fu, Desheng Wen, WaiLun LO

**Affiliations:** 1 Xi’an Institute of Optics and Precision Mechanics of CAS, Xi’an 710119, China; libin@opt.cn (B.L.); ven@opt.ac.cn (D.W.); 2 University of Chinese Academy of Sciences, Beijing 100049, China; 3 Department of Computer Science, Chu Hai College of Higher Education, Tuen Mun, Hong Kong, China; wllo@chuhai.edu.hk

**Keywords:** gaze tracking, infrared camera sensor, near-eye viewing device, CNNs, mobile eye tracker

## Abstract

Eye tracking technology has become increasingly important for psychological analysis, medical diagnosis, driver assistance systems, and many other applications. Various gaze-tracking models have been established by previous researchers. However, there is currently no near-eye display system with accurate gaze-tracking performance and a convenient user experience. In this paper, we constructed a complete prototype of the mobile gaze-tracking system ‘*Etracker*’ with a near-eye viewing device for human gaze tracking. We proposed a combined gaze-tracking algorithm. In this algorithm, the convolutional neural network is used to remove blinking images and predict coarse gaze position, and then a geometric model is defined for accurate human gaze tracking. Moreover, we proposed using the mean value of gazes to resolve pupil center changes caused by nystagmus in calibration algorithms, so that an individual user only needs to calibrate it the first time, which makes our system more convenient. The experiments on gaze data from 26 participants show that the eye center detection accuracy is 98% and *Etracker* can provide an average gaze accuracy of 0.53° at a rate of 30–60 Hz.

## 1. Introduction

In recent years, eye tracking has become an important research topic in computer vision and pattern recognition, because the human gaze positions are essential information for many applications including human–computer interaction (HCI) [1,2], driver assistance [3], optometry, market data analysis, and medical diagnosis. There have been studies using human eye movements for strabismus examination [4]. Parkinson’s disease can also be detected on the basis of human eye blinking. In our previous work [5], we proposed diagnosing developmental coordination disorders (DCD) by detecting changes in patients’ gaze positions and body motion.

The fundamental issues of gaze-tracking technology include tracking system, tracking algorithms, and user experiences. These three issues are closely related to each other. A tracking system may consist of eye cameras, display devices or front-facing cameras, and processing units. Tracking algorithms may include eye region detection, gaze position detection, gaze mapping algorithms, and calibration algorithms, depending on the tracking system. User experiences are highly dependent on the tracking systems and algorithms. Most of the current gaze-tracking systems are either table-mounted or mobile systems. A table-mounted system usually works with an external display screen, which makes human–computer interaction convenient, but it is not robust to head movement. A mobile system is robust to significant head movements, but it is not easy to conduct human–computer interaction with its front-facing camera. A near-eye viewing system combines the advantages of both. To the best of our knowledge, there is no complete prototype for such a system. The fundamental problem of tracking algorithms is to track human eye regions and gaze positions accurately. The cameras are sensitive to light variations and shooting distance, which makes the human eyes very eccentric in the recorded images. In addition, illumination changes, blinking, eyelids, and eyelashes make accurate gaze tracking very challenging. A robust gaze-tracking algorithm is supposed to have stable performance in different environments and efficiently meet the needs of various applications. From the perspective of user experience, the current gaze-tracking systems still have some problems, such as being inconvenient to use, occluding participants’ field of view, a complicated operator interface, and the need to calibrate it before every use. Moreover, most of the current commercial systems are quite expensive. These factors limit the applications of gaze-tracking technology in various research topics.

Regarding the above issues, we propose a mobile gaze-tracking system with a near-eye viewing device, reliable tracking algorithms, and a convenient user experience. The main contributions of our research compared to previous works are as follows.

- We create a complete prototype of an efficient, easy-to-use, and inexpensive mobile gaze-tracking system, *Etracker*. Compared to existing gaze-tracking systems [6,7,8,9], *Etracker* is small, lightweight, unobtrusive, and user-friendly. It records eye movements and computes gaze positions in real time.
- We use a novel near-eye viewing device in the gaze-tracking system, which replaces the traditional large display devices, e.g., computer monitors, TVs, and projectors. The near-eye viewing device has a millimeter-sized display chip with a resolution of 1024×720 pixels and displays a size of 35 × 25 cm^2^ virtual image at a distance of 0.5 m from the human eyes.
- We propose a combined gaze estimation method based on CNNs (ResNet-101) and a geometric model. The CNN is used to remove the blinking eye images and locate the coarse gaze position, and the accurate gaze positions are detected by a geometric model. The gaze accuracy can reach 0.53°.
- We propose using the mean value of pupil centers to smooth the changes caused by nystagmus in calibration algorithms. Therefore, an individual user only needs to calibrate it the first time.

The rest of this paper is organized as follows. In Section 2, a review of previous works on gaze-tracking systems and eye detection algorithms are presented. The proposed gaze-tracking device and algorithms are described in detail in Section 3. In Section 4, the analysis and explanations of the results are presented. Finally, some discussions and conclusions are drawn in Section 5.

## 2. Related Work

Human eye gaze-tracking systems have been used in many fields and researchers have proposed many methods for their detection. Our works are related to previous research on gaze-tracking systems and image-based eye detection methods.

### 2.1. Gaze-Tracking System

The state of the art of gaze-tracking devices can be divided into two main categories: table-mounted gaze-tracking systems and mobile gaze-tracking systems.

Table-mounted gaze-tracking systems are typically designed according to high-resolution visible or infrared cameras and placed at a distance of more than 0.5 m from the participant’s head to record face or eye images. Su et al. [10] proposed an eye–mouse system that allowed people to control computers using their eye movements. They detected the participant’s face and eyes based on a color model and edge features. Lee et al. [11] described a remote gaze-tracking system for large displays (e.g., TVs). They used a wide-view camera for detecting eye position and a narrow-view camera for capturing details. Naqvi et al. [12] proposed a corneal reflection-based gaze-tracking system for assisted driving. They installed an infrared camera in the vicinity of the car dashboard, and used Dlib [13] with three CNNs to extract the left eye, right eye, and face features to estimate the participant’s gaze. In Asier’s research [1], a remote human–computer interaction based on the iPad and its front camera was established. They used a camera to capture the tester’s facial image and built a gaze-tracking model whereby the tester could control the iPad’s applications by moving their eyes or head. Kim et al. [14] built an image acquisition system that is used to distinguish between open and closed human eyes, and they trained a high-precision eye detection model based on CNNs. Krafka et al. [15] used mobile phones to capture the face images of different testers and located the human eye region and gaze by CNNs. Because they collected face images from multiple devices in various orientations, they also designed a unified prediction space to train an end-to-end model using these images. Liu et al. [2] presented a real-time learning attention feedback system that could measure students’ learning attention in unsupervised learning environments. They established a three-layer image processing system to perform motion detection, eye feature extraction, and student learning status analysis. Finally, SVM was utilized to classify the level of learning status. Cerrolaza et al. [16] proposed a taxonomic classification method, which helped researchers to optimize the calibration process for a real VOG gaze-tracking system. Tawari et al. [17] presented a distributed camera framework for driver’s gaze zone estimation by using head pose dynamics, which can operate robustly and continuously even during large head movements. Jung and Jung et al. [18] used an ultrasonic sensor for pupil detection and gaze tracking that is robust to the natural head movements of the user. Pan et al. [19] introduced an optimal gaze-tracking system based on a web camera and ultrasonic sensor, which can accurately measure the user’s head movement. Vora et al. [20] proposed using CNNs to classify the driver’s gaze into seven zones.

Compared with the table-mounted gaze-tracking system, mobile systems can effectively avoid computing the movement between the gaze-tracking device and the participant’s head, which greatly reduces the complexity of the gaze-tracking algorithm and makes the system more robust to head movement. Mobile gaze-tracking systems generally consist of a helmet or glasses that mount the light sources, IR sensors, or near-eye cameras. Kocejko et al. [8] used two web cameras for detecting the positions of the tester’s pupils and head relative to the screen. They wanted to create a user-friendly human–computer interface for people with disabilities. Galante et al. [21] developed a communication assistance system based on gaze-tracking technology to help cerebral palsy patients choose texts and symbols, which can help them create phrases for their daily needs. Gwon et al. [6] presented a mobile gaze-tracking system that can be used by subjects who wear glasses. They counted the number of white pixels in an image that was captured by a low-exposure camera to determine whether the tester wears glasses, then four illuminators were turned on and off sequentially and the gaze point was calculated based on the corneal specular reflections (SR). Kassner et al. [9] showed a lightweight eye-tracking headset and designed an open-source platform for pervasive eye tracking and gaze-based interaction. In [22,23], the authors used a 3D eye model based on the location of the corners and the iris was detected on the unwrapped image by a three-step robust circle-fitting procedure. Borsato et al. [24] used mice chips as imaging sensors and recorded 2D eye-tracking data. Topal et al. [25] proposed a low-computational approach on gaze estimation, which was based on light reflection. They used multiple sensors to record eye images and estimated gaze positions based on least-squares algorithms. Tonsen et al. [26] presented a gaze-tracking method that used four low-resolution cameras to acquire eye images and combined those images based on a machine learning algorithm. Then the gaze positions were achieved according to a gaze-tracking model. Kocejko and Martin [27] proposed an eye-tracking device based on Google Glass and a low-resolution camera, but the resolution of the near-to-eye display is only 640 × 360 pixels and the user experience is poor. Wang et al. [28] presented a wearable gaze-tracking system and a 2D gaze estimation method based on the pupil-glint vector.

### 2.2. Image-Based Eye Detection Methods

Many approaches for image-based eye detection have been proposed in the past years. The curvature of isophotes was used to design an eye center voting system [29]. In [30], the author proposed a method for eye localization based on regression frameworks. A method based on image gradients and squared dots for pupil detection was presented by Timm et al. [31]. Swirski et al. [32] used the Haar-like features for eye region detection at first, and then ellipse fitting based on edge detection was performed to locate the pupil’s contour and center. Araujo [33] described an Inner Product Detector model and correlation filters for eye localization. Borza et al. [34] presented a fast eye segmentation method that extracted multiple features of the eye region. Fuhl et al. [35] proposed a robust pupil detection method that is based on edge filtering and oriented histograms calculated via the Angular Integral Projection Function.

With the development of new visual features and learning-based algorithms (e.g., SVM, HOG, ANN, CNNs, and regression tree), some researchers are interested in using machine learning algorithms to train a robust and efficient eye detector. Fuhl et al. [36] presented a coarse-to-fine pupil positioning method based on two similar convolutional neural networks, and also proposed sub-regions segment from the downscaled input eye images to decrease computational costs. Amos et al. [37] trained a landmark detector using 12 feature points to describe the eye model. Gou et al. [38] built a cascade regression framework for simultaneous eye localization and eye state estimation. Sharma et al. [39] proposed a histogram of oriented gradients (HOG) in combination with support vector machine (SVM) classifiers to obtain an efficient eye detector. With the development of computer hardware and the popularity of the graphics processing unit (GPU), learning-based methods have become mainstream for eye detection approaches due to their high accuracy and robustness.

Although researchers proposed a series of gaze-tracking systems and algorithms, the gaze accuracy is relatively low and the algorithms are complicated. Most of those systems can only be used in a laboratory environment. With the development of camera sensors and display technologies, some companies have developed commercial gaze-tracking devices, as shown in Table 1. The commercial devices shown in Table 1 have higher gaze accuracy than the systems described in [24,26,27], but they are too expensive. Although most research teams are using the above devices, the price factor has still limited the wider applications of gaze-tracking systems. More importantly, there is no complete gaze-tracking system with near-eye display that is not only robust to head movement but also capable of hosting human–computer interaction. Therefore, we propose developing a mobile gaze-tracking system with a near-eye viewing device, reliable tracking algorithms, and a convenient user experience.

## 3. The Proposed Method

### 3.1. Etracker Hardware System and Experimental Environment

**Figure 1:**
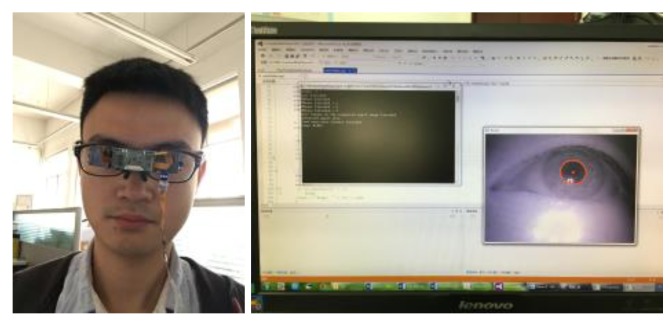
The proposed system and experimental environment.

**Figure 2:**
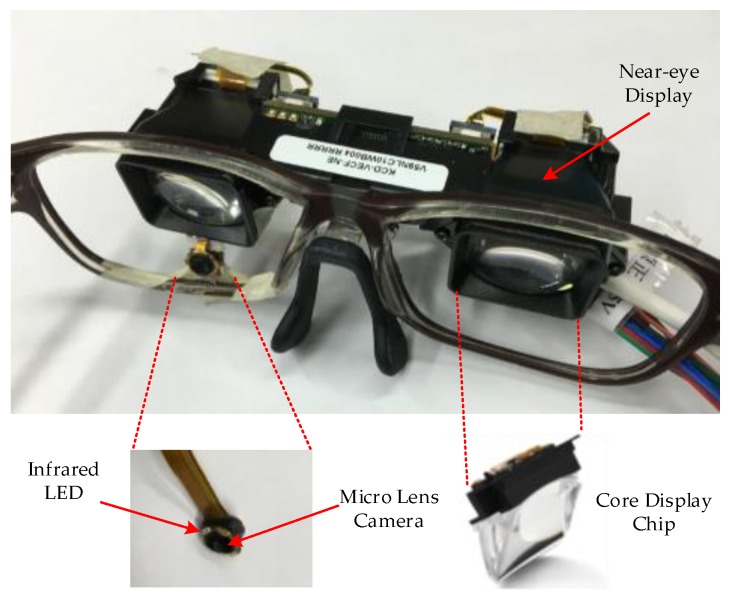
Gaze-tracking device and near-eye viewing device.

Our goal was to design a lightweight and convenient gaze-tracking system. *Etracker*, the proposed gaze-tracking system consists of a micro lens camera and a near-eye viewing device, as shown in Figure 1 and Figure 2. The micro lens camera and the near-eye viewing device are installed on lightweight eyeglasses. The design can not only reduce the device size and weight, but also avoid the occlusions within the participant’s field of view caused by a near-eye camera. The size of micro lens camera is 0.5 cm×0.5 cm×0.3 cm. The resolution is 640×480 pixels and the frame rate is 60 f/s. The size of *Etracker* is 12 cm×4 cm×3 cm and the weight is 52 g.

Compared to a visible light camera, an infrared camera can obtain clearer eye contours and is insensitive to external light changes. That is the reason why we chose an infrared camera to capture eye images. We modified a commercial visible light camera for medical endoscopes as the eye camera. We used a hole punch to cut one round piece of exposed film and fixed it on the camera lens as the infrared filter. Then, six infrared LEDs with 850 nm wavelength were soldered onto the circuit board around the camera. We successfully used this camera to collect eye data from different participants and stored those eye images in computer via the USB3.0 interface. As shown in Figure 2, this tiny micro lens camera makes our system small, lightweight, and user-friendly.

**Figure 3:**
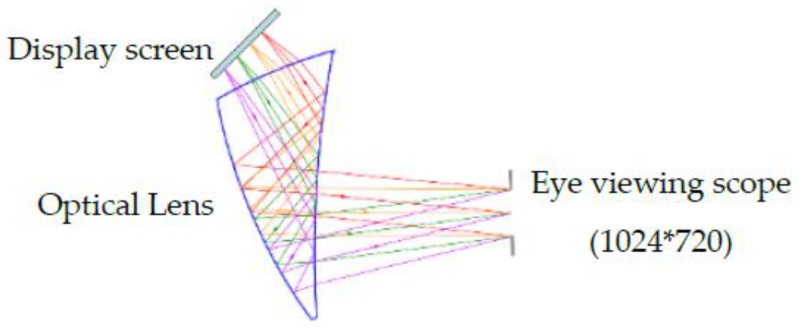
Near-eye viewing device.

Traditional gaze-tracking systems usually require computer monitors or TVs to display the calibrations and gaze information. This makes the gaze-tracking system larger and inconvenient to use. Therefore, the lightweight near-eye viewing technology can greatly reduce the size of the gaze-tracking system. The near-eye viewing device is composed of the OLED micro-display and optic/display holder. It allows the single-element optical design to deliver the nearly 40° field of view as shown in Figure 3. In our gaze-tracking system, the resolution of the near-eye viewing device can reach 1024×720 pixels. The near-eye viewing device can also be easily connected to the computer and embedded devices (e.g., ARM, FPGA, MCU) via the VGA port. The characteristics of the display device used in our system are shown in Table 2. When the participant wears the *Etracker*, the near-eye viewing device will display a series of visual content, e.g., calibration marks, advertisement images, web design photos, videos. The eye camera records the participant’s eye movement in real time. In our experiment, we used an Intel(R) Core(TM) i5-6600 desktop computer with 16 GB RAM and NVIDIA GeForce GTX 745 GPU to collect and process the recorded data.

### 3.2. Workflow of Proposed Gaze-Tracking Method

**Figure 4:**
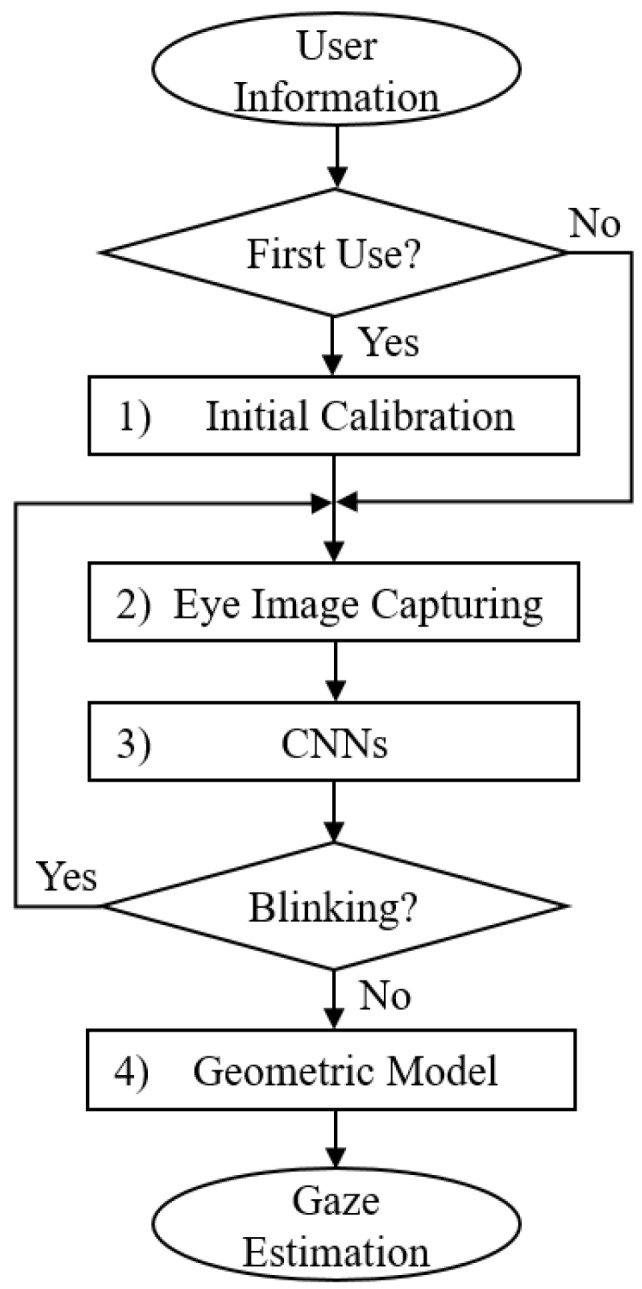
Workflow of the proposed gaze-tracking method.

The overall workflow for the proposed gaze-tracking method is shown in Figure 4. If the user is new to the system, an initial calibration is required. During the calibration, the participant must keep gazing at the flashing mark in the near-eye viewing system until it disappears. If the user did the calibration before, calibration is not needed and it goes straight to the second step. In the second step, we use a single micro lens infrared camera to capture the participant’s eye images in real time, as shown in Figure 1. Then, based on the participant’s eye images recorded during the calibration step, we employed the CNNs model to locate the coarse gaze positions. In this step, we also used CNNs to remove the error images caused by blinking. In the fourth step, the geometric mapping model between the human eyes and the near-eye viewing device was calculated based on the geometric relationship between the participant’s eye locations and gaze positions. Finally, we realized accurate gaze-tracking based on CNNs and geometric model. In the following sections, we will introduce the proposed method in detail.

### 3.3. Initial Calibration

**Figure 5:**
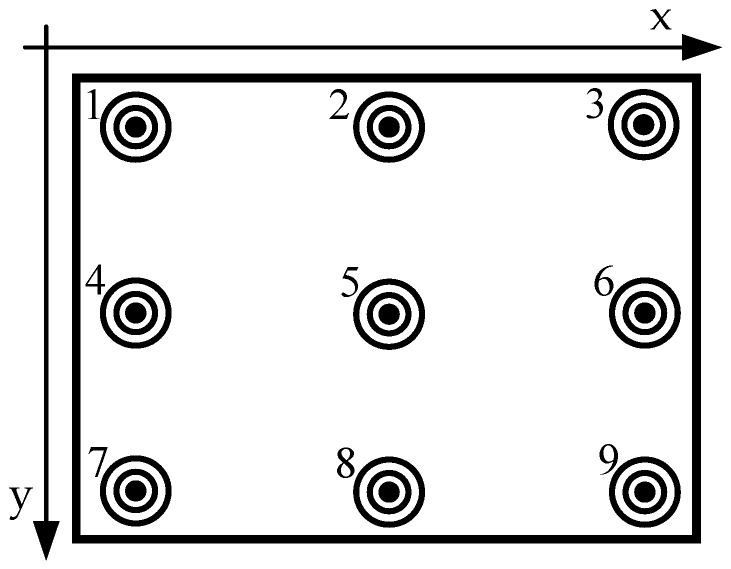
Nine-point calibration marks.

**Figure 6:**
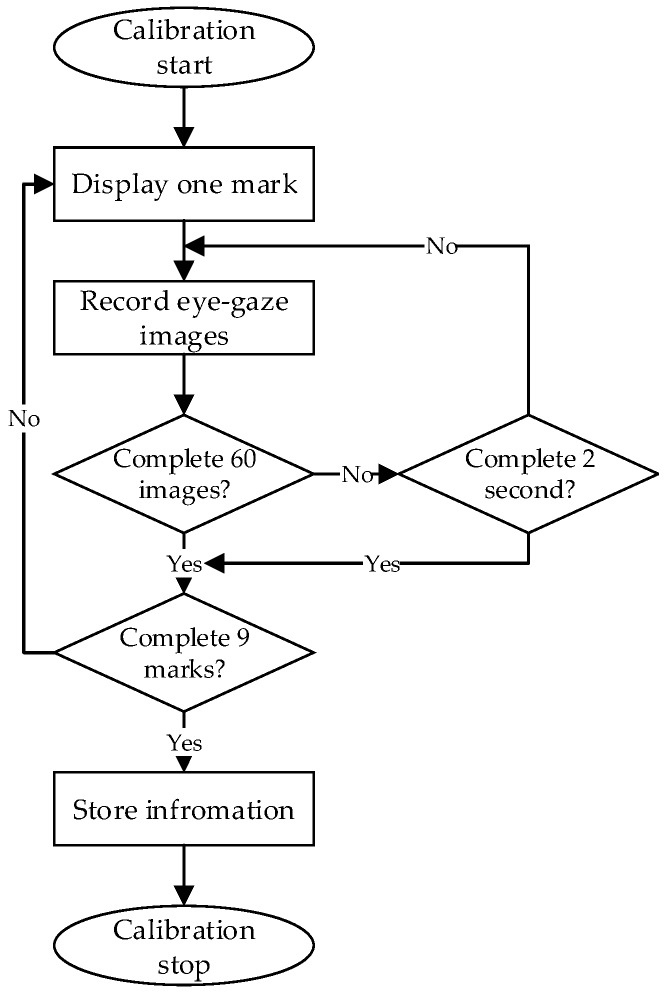
Initial calibration step.

The proposed gaze-tracking system will determine whether the participants need initial calibration based on their input information. The purpose of the initial calibration is to make *Etracker* learn the characteristics of the participant’s eye movements, which helps the proposed gaze-tracking system accurately estimate the participant’s gaze positions. In terms of the number of calibration marks, we have different calibration schemes, such as three-marks, nine-marks, and so on. Considering the calibration speed and accuracy, we adapted nine calibration marks scheme in this paper. As shown in Figure 5, the position of the nine calibration marks on the screen is (112, 60), (512, 60), (924, 60), (112, 360), (512, 360), (924, 360), (112, 660), (512, 660) and (924, 660), respectively. Each calibration mark is comprised of one black inner circle (radius: 5 pixels), one black middle circle (radius: 10 pixels), and one black outer circle (radius: 15 pixels). If the participant needs an initial calibration, the nine calibration marks will flash in the near-eye viewing device randomly. Figure 6 shows the initial calibration step in detail. During the calibration, each mark stays in the display for two seconds, and the participant must keep staring at the mark until it disappears. The infrared camera records the participant’s eye movements at different gaze positions. In order to ensure that the participant concentrates on the calibration task and fixates on display marks accurately, we tested the system in a quiet experiment. For each calibration mark, we collected 60 eye gaze images.

### 3.4. Coarse Gaze Estimation and Blinking Image Removal by CNNs

In our research, we recorded the participant’s eye images through *Etracker*. Then, the coarse gaze positions were estimated based on convolutional neural networks (CNNs). In [29], the authors used an artificial neural network (ANN) and trained a gaze detection function that could directly locate gaze positions from a series of input eye images. Although they obtained some gaze-tracking results through experiments, the gaze estimation model trained in this way is inaccurate in some cases. For example, blinking will lead to false eye gaze prediction. In addition, they needed to train a separate model for each participant. Therefore, we hope to establish a coarse gaze estimation model based on initial calibration data, and use this model to remove error eye images such as blinking.

Given the recent success of ResNet-101 [40] for image recognition, we fine-tuned this model for eye detection and gaze estimation. The main reason we used a pre-trained CNNs model in our work is that training a deep neural network requires a large number of datasets. Using the existing fine-tuned network structure allowed us to achieve better detection results in our dataset. The architecture of the proposed CNNs is summarized in Table 3. The size of the various layers is similar to those of ResNet-101. Because our goal is to design a CNNs that can use the information from a single eye image to efficiently and accurately predict eye status and coarse gaze positions, the number of output nodes in full connect layer is 10. The nine outputs of the softmax layer represent the nine coarse gaze positions and the remaining one output indicates that the eye status is blinking.

A set of eye images associate with ground truth gaze positions collected in the calibration step are used to fine-tune the CNNs. Then we performed eye status detection and gaze estimation based on computations within the CNNs model. Note that in order to use ResNet-101 model through fine-tuning, we normalized the collected eye images (640×480 pixels) to 224 × 224 pixels.

The structure of the CNNs model is shown in Table 3. Conv-1–Conv-5 are the convolutional layers. They are iterated according to the ‘Iterations Number’ shown in Table 3. The max pool and average pool are subsampling layers that can reduce the number of CNNs’ parameters. A full connected layer integrates the feature information after a number of convolutions and pooling operations. Finally, the gaze estimation results are output by the softmax layer.

### 3.5. Combined Gaze-Tracking Algorithm

**Figure 7:**
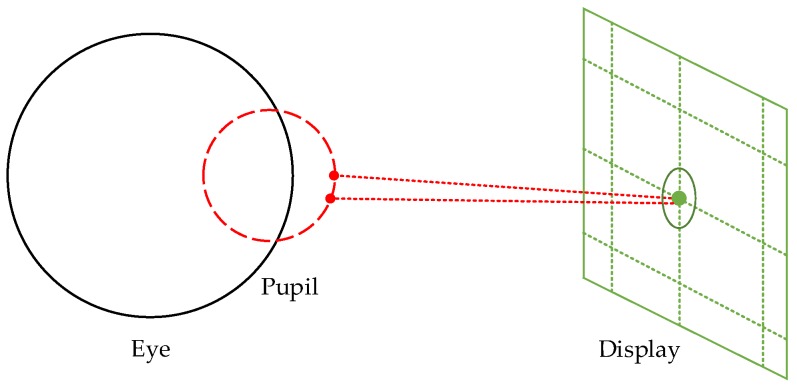
Coarse gaze position estimated by CNNs.

After estimating the coarse gaze positions, we aimed to develop a geometric model between the eye image and near-eye viewing device that can accurately and efficiently calculate the gaze positions. In Section 3.3, we explained that participants need to perform initial calibration when using *Etracker* for the first time. According to the calibration data, we used the CNNs to locate the coarse gaze positions and omit the eye images with blinking. Next, we established an accurate gaze-tracking model based on the geometric relationship between eye positions and gaze points. As shown in Figure 7, the black circle represents the human eye model, the red circle represents the pupil position at different gaze positions, and the green circle and point show the range of coarse gaze position estimated by the CNNs shown in Section 3.4.

Due to nystagmus and light changes, even if the participant maintains the same gaze position, the eye centers in the continuously recorded eye images are different. Using only CNNs for accurate gaze tracking requires a large amount of training data and a long training time. Therefore, when performing the initial calibration step, we collected multiple eye images and used our previous work [41] to detect eye centers $C_{i}\left(x_{j},y_{j}\right)$. Then, we counted the mean value $MC_{i}\left(x,y\right)$ of these eye center positions, where *i* represents the $i^{th}\left(i=1,\ldots ,9\right)$ calibration mark, and *j* denotes the $j^{th}\left(j=1,\ldots ,60\right)$ eye image. We also used the threshold *T* to remove the points that have large deviations from the mean value, where *T* is the Euclidean distance from $C_{i}\left(x_{j},y_{j}\right)$ to $MC_{i}\left(x,y\right)$:

(1) $$if\;C_{i}\left(x_{j},y_{j}\right)-MC_{i}\left(x,y\right)>T\;omit\;else\;C_{i}{}^{\prime }\left(x_{m},y_{m}\right)=C_{i}\left(x_{j},y_{j}\right).$$

**Figure 8:**
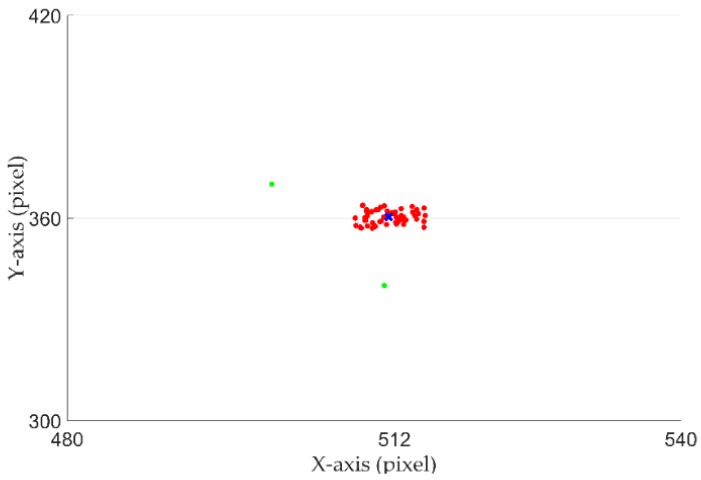
Eye center positions when the participant gazed at the same calibration mark.

Finally, we recalculated the mean value $MC_{i}{}^{\prime }\left(x,y\right)$ of the remaining eye centers $C_{i}{}^{\prime }\left(x_{m},y_{m}\right)$. As shown in Figure 8, the red circles represent the eye centers when the participant gazed at the same calibration mark. The green circles represent misdetection of the eye center due to blinking and the blue cross represents the geometric center, which is calculated by the coordinates’ mean value.

**Figure 9:**
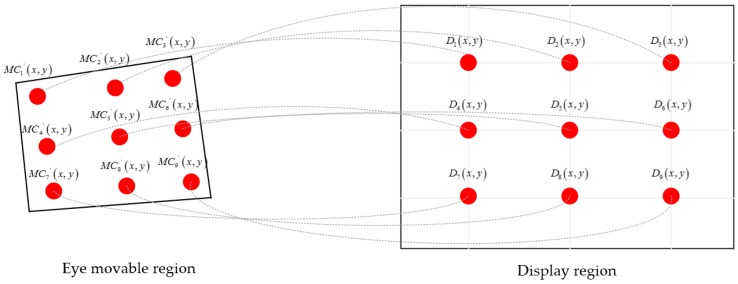
The geometric model of human eye centers and coarse gaze positions.

Figure 9 shows the geometric model between human eye centers and coarse gaze positions; we established an accurate gaze-tracking equation based on their geometric relations. The red circles in the ‘eye moveable region’ represent the geometric eye centers. The red circles in the ‘display region’ represent the coarse gaze positions of CNNs output. According to the structural characteristics of human eyes, when a participant observes an object, the eyes have a larger field of view in the horizontal direction than in the vertical direction. Hence, the distance moved in the horizontal direction is greater than in the vertical direction. We established accurate gaze-tracking equations for the horizontal and vertical directions. The equations are as follows:

(2) $$\begin{array}{l} D(x)=a\cdot MC_{i}{}^{\prime }{\left(x\right)}^{2}+b\cdot MC_{i}{}^{\prime }{\left(y\right)}^{2}+c\cdot MC_{i}{}^{\prime }\left(x\right)+d\cdot MC_{i}{}^{\prime }(y)+e\cdot MC_{i}{}^{\prime }(x)\cdot MC_{i}{}^{\prime }(y)+f\\ D(y)=g\cdot MC_{i}{}^{\prime }{\left(x\right)}^{2}+h\cdot MC_{i}{}^{\prime }{\left(y\right)}^{2}+i\cdot MC_{i}{}^{\prime }(x)+j\cdot MC_{i}{}^{\prime }(y)+k\cdot MC_{i}{}^{\prime }(x)\cdot MC_{i}{}^{\prime }(y)+l \end{array},$$

where $MC_{i}{}^{\prime }\left(x,y\right)(i=1,\ldots ,9)$ is the eye position and $D_{i}\left(x,y\right)$ is the coarse gaze position. Then, we used the coordinate relationship between the eye centers and coarse gaze positions to calculate the equations’ parameters, i.e., *a*, *b*, *c*, …, *l*. Finally, we only needed to detect the eye centers and use the geometric model to realize the participant’s gaze tracking accurately and automatically.

## 4. Results

### 4.1. Dataset Collection

**Figure 10:**
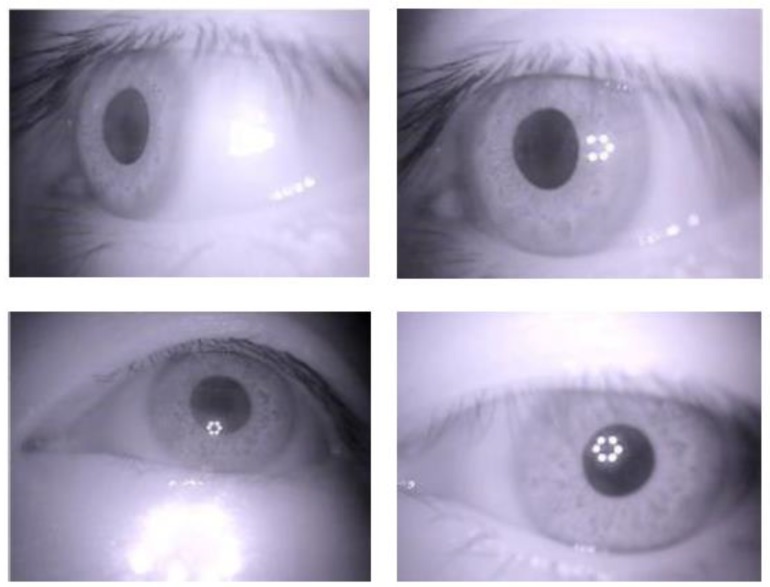
Samples of images in the dataset.

To verify the stability and performance of *Etracker*, we collected 26 participants’ data in a challenging real-word environment. The participants were aged between 20 and 29 years and included both males and females. Each participant needed to perform five repetitions of tests, including one calibration test, two non-calibration tests to generate the training dataset, and two other non-calibration tests to generate the testing datasets. The participants were asked to take the *Etracker* off and put it back on after each test. Some sample images are shown in Figure 10.

### 4.2. Gaze Tracking Results

**Figure 11:**
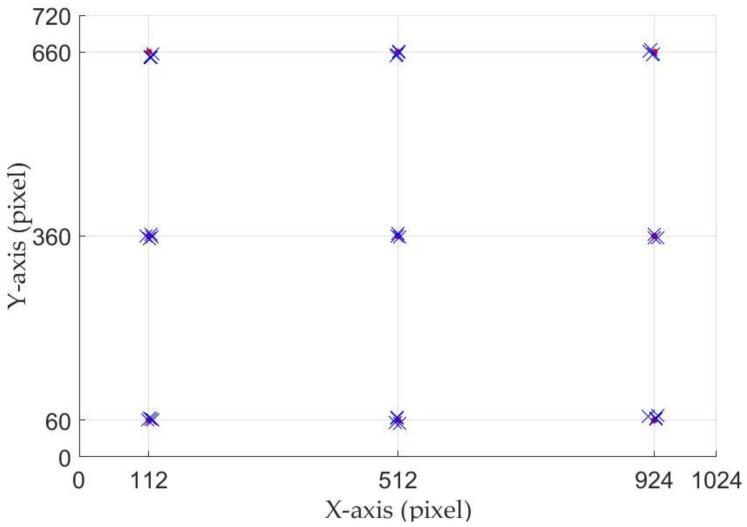
Estimated gaze positions based on nine calibration marks.

In our experiments, a gaze-tracking model was established after each participant first used *Etracker*. Then the participants repeatedly gazed at the calibration marks four times. The established gaze-tracking model was used to predict the participant’s gaze positions. Some examples are shown in Figure 11. The red circles represent the calibration marks and the blue crosses are the gaze positions estimated by the *Etracker*. The results show that there are small errors of distance between the predicted gaze positions and the ground truth values.

**Figure 12:**
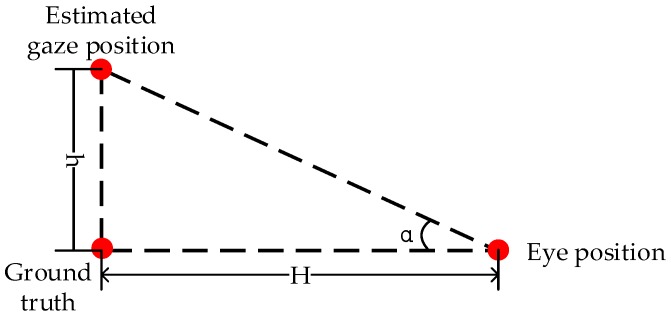
Error calculation model.

In order to further illustrate the accuracy of the proposed gaze-tracking system, we use $\alpha^{\circ }$ to measure the error between the estimated gaze position and ground truth as shown in Figure 12 and Equation (3). The distance from the images displayed in the near-eye viewing device to the participant’s eye is about H = 50 cm.

(3) $$\tan\alpha^{\circ }=\frac{h}{H}$$

We randomly selected eight participants from our dataset. Table 4 and Table 5 show the accuracy of gaze estimation by only CNNs and the combined gaze-tracking algorithm, respectively. The results show that the *Etracker* gaze accuracy is approximately 0.74°, using only CNNs for gaze estimation. If we used the combined gaze-tracking algorithm, i.e., CNNs and geometrical model, the gaze accuracy can be significantly improved to around 0.54°. We also notice that for the CNNs method gaze position 5 is more accurate than gaze positions 1, 3, 7, and 9 (Table 4). Because gaze position 5 is at the center of the near-eye viewing device, the participant’s pupil has smaller distortion when they gaze at position 5.

We also calculated the error of estimated gaze positions in the horizontal and vertical directions in Table 6. The results show that the gaze accuracy in the horizontal direction is higher than in the vertical direction. Because our infrared camera captures images below the human eyes (in Figure 2), the horizontal displacements are easier to detect than vertical displacements.

As shown in Table 7, we randomly selected eight participants to perform the initial calibration, and then counted the results of repeated use of *Etracker* four times without calibration. We found that, even for multiple tests with different participants, there was no significant increase in the tracking error. The average gaze-tracking error of the proposed device is still around 0.54°. This shows that our proposed system is calibration free once the initial calibration is done.

**Figure 13:**
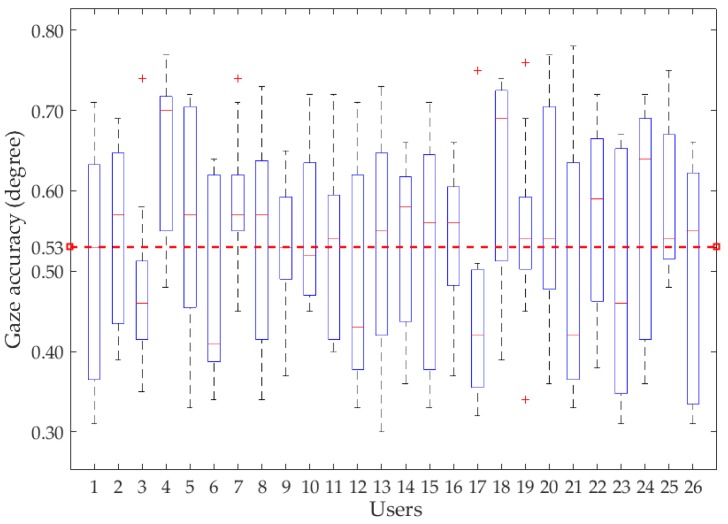
Gaze-tracking accuracy of different participants.

Figure 13 shows the accuracy of 26 participants’ gaze tracking through the combined gaze-tracking algorithm. The results show that the average gaze detection accuracy is approximately 0.53°.

**Figure 14:**
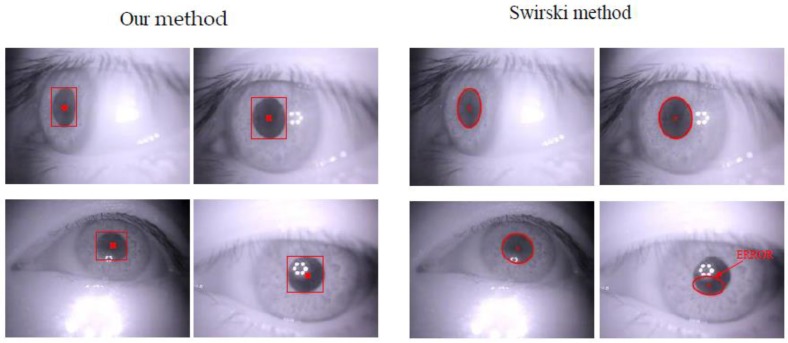
Some results of eye location compared to the state-of-the-art method.

In our research, we built a geometric model to locate the accurate gaze positions based on CNN outputs (Section 3.5). Figure 14 shows some qualitative results of eye center detection using our system and Swirski’s method [32]. Swirski’s approach first performs edge detection, then randomly selects a feature point on the pupil’s edge and uses an elliptic equation to fit the pupil edge and center. However, it fails if the pupil region is affected by external light, e.g., the last image. This proves that our system can successfully detect eye locations even if the pupil region is blurred. The highly precise and robust eye detection algorithm ensures that the geometric model can accurately predict the participant’s gaze positions. In our dataset, the eye centers detection accuracy is 98%.

A comparison between the proposed *Etracker* and the state-of-the-art gaze-tracking devices is presented in Table 8. Note that, even we use only CNNs for coarse gaze position estimation, the tracking accuracy is significantly better than in Mayberry [42], Tonsen [26], and Borsato [24]. Although the tracking speed of our method (60 Hz) is lower than Borsato (1000 Hz), our device is small and highly integrated, which means it can be easily used in practical applications. Compared to the binocular gaze-tracking device proposed by Kassner [9], our system only requires one IR camera and the system cost is lower.

**Figure 15:**
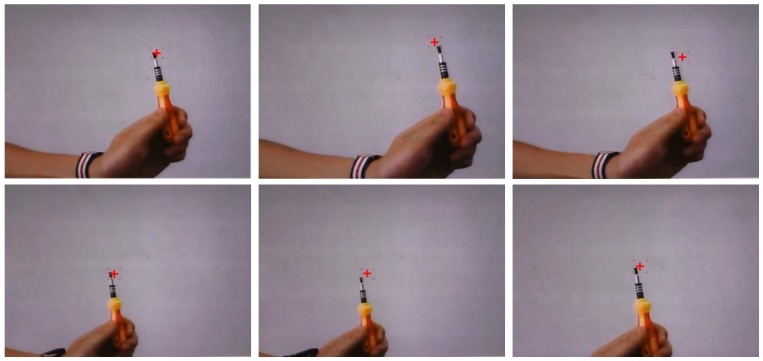
Some results of real-time gaze tracking.

*Etracker* can reach the best tracking accuracy (0.53°) compared to existing gaze-tracking systems, and the gaze-tracking speed can meet daily application requirements. Furthermore, compared with Krafka’s [15] system, in which only rough gaze positions can be detected, our system can realize the tracking of any participant’s gaze positions in real time, as shown in Figure 15. The participant is asked to gaze at the pen tip displayed in the near-eye viewing device. The red cross represents the participant’s gaze position calculated by *Etracker* and the results show that our proposed gaze-tracking system can accurately locate the participant’s gaze position.

### 4.3. Further Discussion

The experimental results demonstrate that our proposed *Etracker* gaze-tracking system can achieve satisfying gaze-tracking accuracy. Eye blinking is an important issue affecting the accuracy of gaze tracking, which leads to incorrect gaze data and makes the absolute orientation unusable. Most current gaze-tracking systems do not take blinking into account; they only verify their devices in an ideal environment. Our proposed system solves this problem very well. In our system, we use the CNNs joint geometric model for accurate gaze tracking. With a CNNs learning-based model, we can not only remove the error images due to eye blinking and nystagmus, but also predict the coarse gaze positions. After the first initial calibration, participants do not need to calibrate it for later use, which makes our gaze-tracking system more user-friendly. In contrast to some existing gaze-tracking devices that can only be used to track the specific gaze position, e.g., nine calibration marks, our system can detect any gaze position within the participant’s field of view in real time.

## 5. Conclusions

In this paper, we proposed a mobile gaze-tracking system, *Etracker*, with a near-eye viewing device. We adopted a low-cost micro lens infrared camera to record a participant’s eye images in real time. The size and cost of the proposed gaze-tracking system is greatly reduced with this design. We do not need to use large-scale monitors or a calibration board during the calibration step; both are needed in traditional gaze-tracking systems.

In order to further improve the accuracy of the proposed gaze-tracking system, we established a combined gaze-tracking model based on CNNs and geometrical features. We performed fine-tuning with a pre-trained ResNet-101, and used this neural network to remove faulty eye images such as blinking and predict coarse gaze positions. In order to locate the gaze positions more accurately, we built a geometrical model based on the positional relationship between the participant’s eye centers and the near-eye viewing device. Coordinates’ mean value was used to locate the stable eye centers, so that only initial calibration was needed for an individual user. The experimental results from 26 participants showed that the proposed gaze-tracking algorithm makes the system’s gaze accuracy reach 0.53°.

In future work, we hope to improve the gaze accuracy of *Etracker* by designing a binocular gaze-tracking system. We also plan to collect more participant data to further improve the accuracy and robustness of the gaze-tracking system.

## Figures and Tables

**Table 1:** Comparison of several commercial gaze-tracking systems.

Device	Tobii Glasses II	PupilLabs	Tobii X2-30	Tobii X2-60
**Category**	Head-mounted	Head-mounted	Table-mounted	Table-mounted
**Sample rate**	50–100 Hz	30–200 Hz	30 Hz	60 Hz
**Gaze Accuracy**	0.5°	0.6°	0.4°	0.4°
**Weight**	45 g	48 g	200 g	200 g
**Calibration**	Yes	Yes	Yes	Yes
**SDK**	Yes	Yes	Yes	Yes
**Connection**	Micro USB	USB Type C	USB2.0	USB2.0
**Price**	30,000 €	1840 €	>10,000€	>30,000 €

**Table 2:** The performance of the near-eye viewing device in *Etracker*.

Resolution	1024 × 720 pixels
Field of View (Horizontal)	31.6°
Field of View (Vertical)	24.2°
Weight	31 g
Device Size	11 × 3 × 3 cm^3^
Core Display Chip size	4.5×4.0×2.7 mm^3^
Connection	VGA

**Table 3:** Structure of CNNs model used in our work.

Layer		Kernel Size	Filters Number	Feature Map Size	Stride	Padding	Iterations Number
**Image Layer**				224×224×3			
**Conv-1**		7×7	64	112×112	2	3×3	1
Max pool		3×3	1	56×56	2	-	1
**Conv-2**	Conv-2-1	1×1	64	56×56	1	0	3
	Conv-2-2	3×3	64		1	1	
	Conv-2-3	1×1	256		1	0	
**Conv-3**	Conv-3-1	1×1	128	28×28	2	0	4
	Conv-3-2	3×3	128		1	1	
	Conv-3-3	1×1	512		1	0	
**Conv-4**	Conv-4-1	1×1	256	14×14	2	0	23
	Conv-4-2	3×3	256		1	1	
	Conv-4-3	1×1	1024		1	0	
**Conv-5**	Conv-5-1	1×1	512	7×7	2	0	3
	Conv-5-2	3×3	512		1	1	
	Conv-5-3	1×1	2048		1	0	
Average pool		1×1	1	7×7	1	0	1
Full Connect layer		10	-	-	-	-	1
Softmax		10	-	-	-	-	1

**Table 4:** The errors in coarse gaze detection when using only the CNNs method (unit: °).

	User	User 1	User 2	User 3	User 4	User 5	User 6	User 7	User 8	Average
	Gaze Point									
**1**		0.78	0.83	0.76	0.87	0.88	0.87	0.81	0.72	0.82
**2**		0.75	0.97	0.60	0.73	0.62	0.63	0.65	0.65	0.70
**3**		0.95	0.81	0.87	0.98	0.67	0.73	0.76	0.69	0.81
**4**		0.53	0.64	0.72	0.52	0.68	0.73	0.65	0.65	0.64
**5**		0.64	0.60	0.53	0.54	0.62	0.63	0.62	0.62	0.60
**6**		0.63	0.51	0.78	0.52	0.63	0.55	0.88	0.65	0.64
**7**		0.80	0.72	0.84	0.87	1.00	0.83	0.74	0.93	0.84
**8**		0.74	0.81	0.77	0.76	0.70	0.73	0.95	0.72	0.77
**9**		0.68	0.94	0.69	0.97	0.88	0.91	1.01	0.97	0.88
**Average**		0.72	0.76	0.73	0.75	0.74	0.73	0.79	0.73	0.74 *

(* It is row average.).

**Table 5:** The errors in accurate gaze detection by combined gaze-tracking algorithm (unit: °).

	User	User 1	User 2	User 3	User 4	User 5	User 6	User 7	User 8	Average
	Gaze Point									
**1**		0.50	0.77	0.42	0.48	0.57	0.34	0.71	0.58	0.55
**2**		0.34	0.55	0.50	0.71	0.45	0.76	0.70	0.61	0.58
**3**		0.42	0.54	0.35	0.31	0.67	0.69	0.62	0.59	0.52
**4**		0.36	0.47	0.37	0.32	0.39	0.54	0.49	0.40	0.42
**5**		0.39	0.75	0.77	0.38	0.64	0.52	0.71	0.45	0.58
**6**		0.42	0.48	0.78	0.62	0.39	0.52	0.57	0.54	0.54
**7**		0.51	0.36	0.59	0.67	0.48	0.45	0.48	0.42	0.50
**8**		0.32	0.69	0.33	0.62	0.61	0.55	0.77	0.72	0.58
**9**		0.75	0.49	0.42	0.53	0.69	0.56	0.74	0.40	0.57
**Average**		0.45	0.57	0.50	0.52	0.54	0.55	0.64	0.52	0.54 *

(* Row average.).

**Table 6:** Gaze tracking errors in horizontal and vertical directions by combined gaze-tracing algorithm.

	Coordinate Error	H	V
	User		
**User 1**		0.48	0.49
**User 2**		0.52	0.54
**User 3**		0.37	0.48
**User 4**		0.48	0.51
**User 5**		0.49	0.58
**User 6**		0.53	0.57
**User 7**		0.59	0.71
**User 8**		0.49	0.53
**Average**		0.49	0.55

**Table 7:** The errors of repeated use of *Etracker* with only one initial calibration (unit: °).

	Coordinate Error	1st	2nd	3rd	4th	Average
	User					
**User 1**		0.45	0.42	0.48	0.47	0.46
**User 2**		0.57	0.54	0.54	0.6	0.56
**User 3**		0.50	0.52	0.49	0.53	0.51
**User 4**		0.52	0.51	0.53	0.55	0.53
**User 5**		0.55	0.49	0.52	0.54	0.53
**User 6**		0.55	0.53	0.56	0.55	0.55
**User 7**		0.64	0.59	0.67	0.58	0.62
**User 8**		0.52	0.53	0.49	0.60	0.54
**Average**		0.54	0.52	0.54	0.55	0.54 *

(* Row average.).

**Table 8:** Comparison with existing gaze-tracking system.

	Normalized Error	Gaze Accuracy (°)	Method	Rate (Hz)
	Method			
**Mayberry** [42]		3	ANN	30
**Tonsen** [26]		1.79	ANN	-
**Borsato** [24]		2.1	Optical Flow	1000
**Kassner** [9]		0.6	Geometric Model	24
**Gwon** [6]		0.7	Corneal Reflection	15
**Ours**		0.75	CNNs	60
**Ours**		0.53	Combined gaze-tracking algorithm	30–60
